# Experimental Approach of Quadriceps Strength Measurement: Implications for Assessments in Critically Ill Survivors

**DOI:** 10.3390/diagnostics12010202

**Published:** 2022-01-14

**Authors:** Anne-Françoise Rousseau, Isabelle Kellens, Pierre Delanaye, Olivier Bruyère, Benoit Misset, Jean-Louis Croisier

**Affiliations:** 1 Intensive Care Department and Burn Centre, University Hospital, University of Liège, 4000 Liège, Belgium; isabelle.kellens@chuliege.be (I.K.); benoit.misset@chuliege.be (B.M.); 2 Department of Nephrology-Dialysis-Transplantation, University Hospital, University of Liège, 4000 Liège, Belgium; pdelanaye@chuliege.be; 3 Department of Nephrology-Dialysis-Apheresis, Hôpital Universitaire Carémeau, 30900 Nîmes, France; 4 Department of Public Health, Epidemiology and Health Economics, University of Liège, 4000 Liège, Belgium; Olivier.Bruyere@uliege.be; 5 Department of Sport Sciences and Rehabilitation, University of Liège, 4000 Liège, Belgium; jlcroisier@uliege.be

**Keywords:** quadriceps strength, dynamometry, test conditions, measurement bias, reliability

## Abstract

(1) Background: The supine testing position is suitable for early quadriceps strength (QS) assessment in intensive care unit, while a seated position is more appropriate for survivors who have regained mobility. Acquiring consistent measurements is essential for longitudinal follow-up. We compared the QS generated in different settings in healthy volunteers. (2) Methods: Isometric QS was assessed using a MicroFet2 and standardised protocols comparing different modalities. Hip and knee flexion angles were, respectively, 45° and 40° (H45-K40) in the supine position, and both at 90° (H90-K90) in the seated position. Dynamometer was either handheld (non-fixed configuration, NFC), or fixed (FC) in a cubicle. (3) Results: QS in H90–K90 and H45-K40 positions were strongly correlated, but QS was higher in the later position regardless of the configuration. Compared to H45-K40, biases of 108.2N (or 28.05%) and 110.3N (27.13%) were observed in H90-K90 position, respectively, in the NFC and FC. These biases were independently and positively associated with QS (*p* < 0.001). For both position, there were no significant differences between QS measured in NFC or FC. (4) Conclusions: The quadriceps was less efficient in the seated position, compared to the supine position, in healthy volunteers. These findings have practical implications for further assessments and research in critically ill patients.

## 1. Introduction

The long-term consequences of critically ill survivors have become an increasing public health concern. Muscle weakness is common in critically ill patients who survive a stay in an intensive care unit (ICU) [1] and has been associated with prolonged ICU and hospital stay, prolonged duration of mechanical ventilation, altered quality of life, increased health-related costs and both acute and long-term increased mortality [2]. In this context, measuring muscle strength early is important to identify the weakest patients and decide on the most appropriate interventions. Measuring of muscle strength is also relevant throughout the post-ICU trajectory, to monitor the recovery.

Several methods aiming to assess muscle strength are described. Manual muscle testing using the Medical Research Council (MRC) scale is still the most used tool to diagnose ICU-acquired weakness (ICU-AW) [2,3,4,5]. Manual testing is subjective and doubts remains about its suitability as a screening tool due to its limited sensitivity at higher grades (MRC grades 4 and 5 are not easily differentiated and progress in muscle strength is not easily detected when applied to stronger muscle groups) [6,7]. Dynamometry is a more objective alternative and one of the most accurate clinimetric tool to assess muscle strength [3]. It can be performed as early during patient care, as long the patient is cooperative. Handgrip dynamometry has become another standard for ICU-AW diagnosis, and cut-off scores have been described for both men and women [8,9]. However, handgrip dynamometry may not be suitable to characterise lower limb strength and related functional activities [7,10,11]. The quadriceps is essential for standing, sitting, and walking, so its strength has been correlated to lower limb function [12,13,14]. This makes quadriceps strength a relevant physical outcome to measure.

A well-defined test position is critical for the reliability and reproducibility of muscle strength measurement. In many studies reporting quadriceps strength in the ICU [15] or other settings [16,17,18], patients were in seated position with the tested leg at 90° of hip flexion and 90° of knee flexion. This position is not suitable for early assessment of critical patients but is rather appropriate for ICU survivors who have regained some level of mobility and autonomy. On the contrary, the supine position is easily feasible during ICU stay. We recently proposed and validated a highly standardised protocol of quadriceps strength measurement in a modified supine position with the tested leg at 45° of hip flexion and 40° of knee flexion [19,20]. However, due to the required equipment, it is more difficult to use this protocol outside of the ICU, such as in a post-ICU follow-up consultation. In addition to the leg position, the dynamometer configuration also needs to be defined: hand-held or fixed on a support. If the first technique is often used in clinical practice, the second requires more equipment but is independent of the examiner’s resistive force.

For a longitudinal follow-up of muscle strength from ICU stay to recovery, acquiring consistent and comparable measures is essential, both for clinical and research purposes. Thereby, it is important to clarify if leg position and dynamometer configuration are interchangeable without impacting strength. To the best of our knowledge, effects of leg position and dynamometer conditions on quadriceps performances during isometric contraction have rarely been studied. The aims of the present study were to compare the quadriceps strength generated in two different combinations of hip and knee angles (hip 90° and knee 90°, versus hip 45° and knee 40°) and with two dynamometer configurations (non-fixed or fixed). This pragmatic study was performed on healthy volunteers as a preliminary unavoidable methodological step before exploring the pathological equivalent.

## 2. Materials and Methods

This study was conducted in 2021, after approval by the local Ethics Committee of our University Hospital (National Ref B7072021000006, Local Ref 2021/45, 31 March 2021). The participants were fully informed of the study purpose, procedures, and limited risks prior to enrolment.

### 2.1. Participants

A convenience sample of healthy volunteers was recruited among the medical and paramedical ICU staff members, and among subjects who attended upper limb physiotherapy sessions in our hospital. Inclusion criteria included age ≥ 18 y. Exclusion criteria included total hip or knee arthroplasty in the dominant limb, pre-existing myopathy or polyneuropathy, and a history of traumatic spine or lower limb injury within the past 6 months.

### 2.2. Quadriceps Strength Testing

Maximal isometric voluntary quadriceps contraction was assessed using a handheld dynamometer (MicroFet2**^®^**, Hoggan Health Industries, West Jordan, UT, USA) with a curved transducer pad. The same trained examiner (a physiotherapist) performed all strength measurements. The highly standardised protocol is detailed in a previously published validation study [19]. Intra-observer reliability has been demonstrated in that princeps study including patients with critical illness [19]. The dominant limb was tested, defined as the reported kicking leg [21]. The MicroFET2 was localised at the anterior face of the ankle, two centimetres above external malleolus level. The protocol consisted of three consecutive maximal contractions, preceded by three progressively intensified warm-up trials. Subjects were first shown the movement to be tested (“push against the dynamometer by attempting to perform knee extension”) and then asked to perform it to confirm their understanding and finally did the warm-up. The three measurements were then performed with 30 s intervals between contractions. Subjects were asked to gradually increase their muscle force to a maximum effort that had to be sustained for 6 s. Operator provided standardised encouragements (“Ready! Push! Build it up! Push harder! Harder! Harder! Harder! Stop!—with one order per second) to ensure maximal effort during each trial. As the protocol aimed to give the participant the chance to reach his/her maximal strength and not to test the capacity to repeat a muscular effort, the best performance out of the 3 measurements was considered for the analysis. Muscle strength was expressed in Newton (N). In order to reduce inter-individual variability and minimise the effect of subject weight on muscle strength, absolute strength was normalised according to actual body weight (expressed in N/kg).

### 2.3. Participant Position

**Figure 1:**
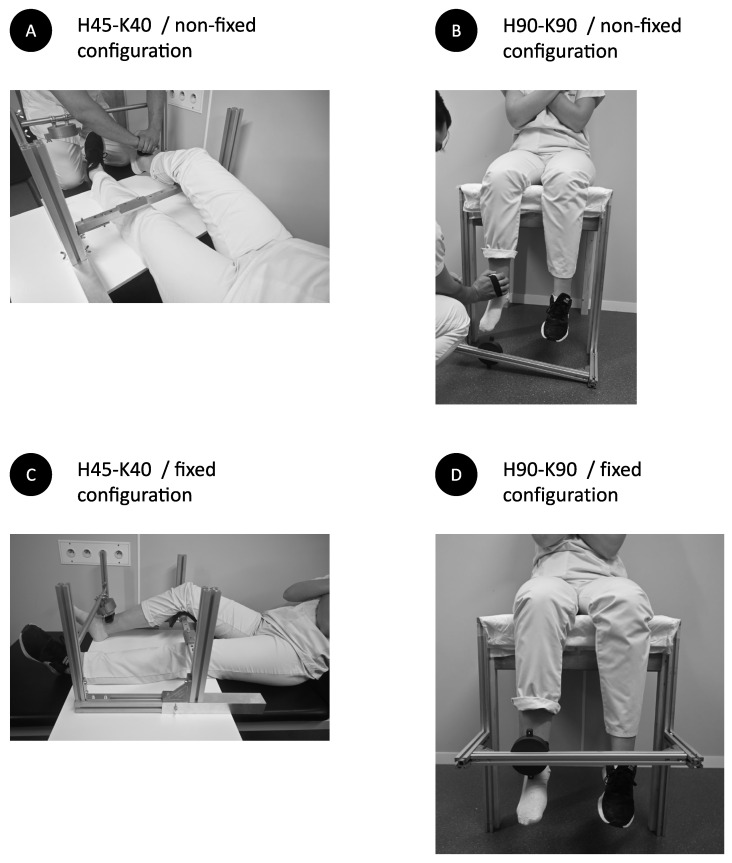
Illustration of the testing structure, participant positioning and operator positioning.

Measurements were performed in two positions, supine and seated positions, with the arms crossed on the thorax. In the supine position (Figure 1A,B), limb position was standardised using an adjustable system of vertical and horizontal bars, aiming to acquire a 45° hip flexion and a 40° knee flexion (H45-K40) and to maintain the mandatory position during all the procedure. For comfort purpose, the back of the knee of the tested limb was resting on a solid cushion, that could not be flattened. In the seated position (Figure 1C,D), thighs rested on a wooden stand without backrest. The legs were hanging, with hip and knee flexed at 90° (H90-K90). Correct lower limb position was confirmed using a long arms goniometer.

### 2.4. Dynamometer Configuration

Measurements were performed in two configurations: non-fixed and fixed dynamometer. In the non-fixed method (Figure 1A,C), the operator was positioned in the front of the participant and held the MicroFET2 in her hands, withstanding the subject’s movement (knee extension). In the fixed method (Figure 1B,D), the MicroFET2 was fixed in a custom-made plastic cubicle, fixed on the front bar. The position of the cubicle was adaptable by sliding it on the front bar, by tilting it on its axis and by moving the front bar on two axis height and depth), in order to position the MicroFET2 at ankle level, as described above.

### 2.5. Testing Repetition

The order of the tests (H45-K40 non-fixed, H45-K40 fixed, H90-K90 non-fixed and H90-K90 fixed) were randomly assigned. The measurement in each setting was conducted with a five-minute interval to prevent muscle fatigue. The same MicroFET2 was used for all tests.

### 2.6. Other Descriptive Data

Age, gender, weight, and height, body mass index (BMI) were recorded. Physical activity status was also characterised according to the participant’s self-report: participants who reported recreational physical activity or sports activity for 4 or more hours per week were considered physically active, while patients who did not achieve this cut-off were considered physically inactive. Discomfort at tested limb was evaluated after each test using a numeric rating scale. Participant was asked to rate discomfort from 0 to 10, understanding that 0 is equal to no discomfort and 10 is equal to the worst possible discomfort.

### 2.7. Analysis

Statistical analysis was performed using Graphpad Prism (version 9.0 for Mac OSX, Graphpad Inc., San Diego, CA, USA). Qualitative parameters were expressed as counts and percentages. Normality was assessed using the Shapiro-Wilk test. As some datasets did not pass the normality test, results were expressed as medians with interquartile ranges [P25 and P75] for quantitative parameters and comparisons were performed using nonparametric tests. Paired data were compared using Wilcoxon test. Unpaired data were compared using Mann-Whitney test. Correlations between measures were tested using Spearman test. Performances of the seated and fixed measurements were compared with the reference method (supine position with non-fixed configuration) using Bland-Altman analysis. The same analysis was used to compare the performances of the fixed configuration with the non-fixed configurations, considered arbitrarily as the reference method. Simple and multiple regression models were established to relate the observed difference between strength generated in the two positions (as independent variable) to quadriceps strength and participant’s demographic parameters (dependent variables). A *p* value < 0.05 was considered statistically significant.

## 3. Results

**Figure 2:**
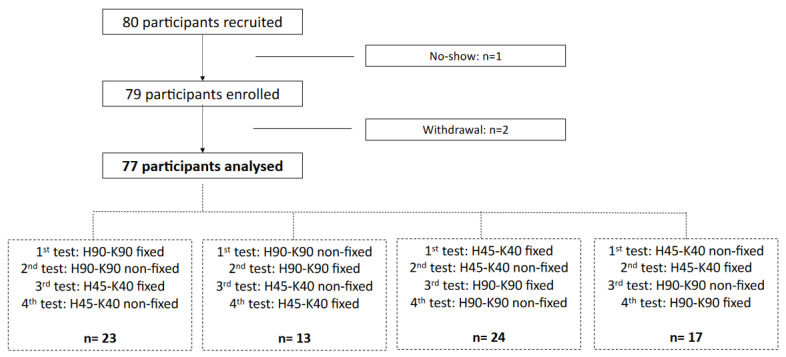
Flow chart, detailing the order of the tests among the included participants.

From the 80 recruited volunteers, 1 subject did not show up for the appointment, and two others gave up the tests due to back pain that preceded the test. Finally, 77 participants (34 males, 44.2%) were analysed (Figure 2). Median age of the subjects was 42 (25–57) years; median height, 170 (164–179) cm; median weight, 75 (64–83) kg; and median body mass index, 24.3 (22.2–27) kg/m^2^. Among the 77 subjects, 52 (67.6%) were physically active.

**Figure 3:**
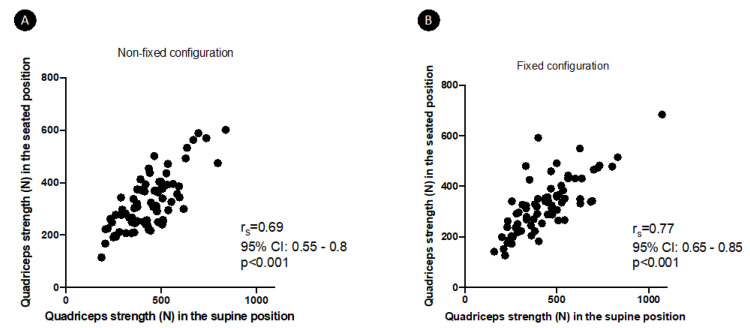
Correlations between quadriceps strength in the seated (H90-K90) and supine (H45-K40) positions, in non-fixed (**A**) and fixed (**B**) configuration.

The measured quadriceps strength in the different settings are shown in Table 1. There was a significant positive correlation between quadriceps strength measured in the H45-K40 position and in the H90-K90 position: r_s_ = 0.69 (95%CI 0.55–0.8, *p* < 0.001) in non-fixed configuration and r_s_ = 0.77 (95%CI 0.65–0.85, *p* < 0.001) in fixed configuration (Figure 3). Strength in the H45-K40 position was significantly higher than in the H90-K90 position (*p* < 0.001 whatever the dynamometer configuration). On the contrary, for a same position, there was no significant difference between strength measured in non-fixed or fixed configuration. Discomfort was rated similarly in the different positions and configurations (Table 1).

**Figure 4:**
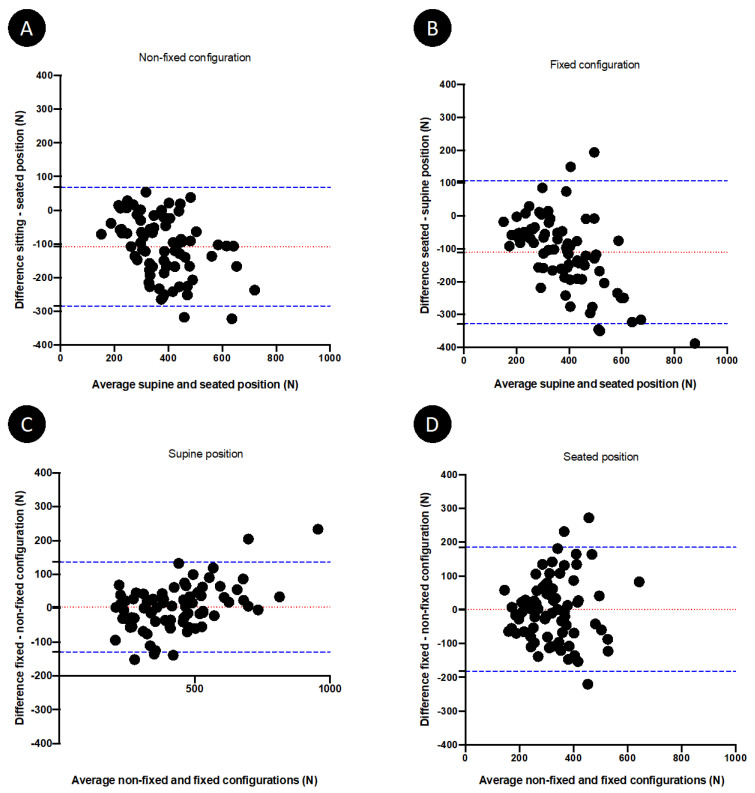
Bland–Altman plots showing the difference between quadriceps strength in the seated (H90-K90) and in the supine (H45-K40) position as the comparison method, in non-fixed (**A**) and fixed (**B**) configuration, and the difference between quadriceps strength in fixed configuration and in non-fixed configuration as the comparison method, in the supine (H45-K40) (**C**) and the seated (H90-K90) (**D**) position.

**Figure 5:**
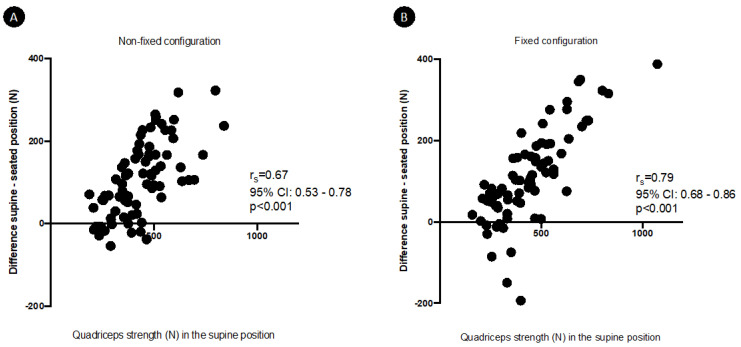
Correlations between quadriceps strength in the supine position and the difference between strength in the seating (H90-K90) and supine (H45-K40) position, in non-fixed (**A**) and fixed (**B**) configuration.

Performances of quadriceps measurements according to the hip and knee position and to the dynamometer configuration are represented in Bland-Altman plots (Figure 4). Biases were demonstrable for the H90-K90 position when compared to the H45-K40 position, considered as the reference method. These biases reached 28.05% (95% confidence interval agreement limit from −71.84 to 15.75%) and 27.13% (95% confidence interval agreement limit from −72.77 to 18.52%) in the H90-K90 position, respectively, in non-fixed and fixed configuration. The mean difference between strength generated in the H45-K40 and H90-K90 positions significantly and strongly correlated with the quadriceps strength in the H45-K40 position, whatever the dynamometer configuration (Figure 5). The results of multiple linear regression showed that this mean difference was influenced by quadriceps strength (*p* < 0.001 whatever the dynamometer configuration), independently of age, sex, BMI and physical activity. Similarly, the percentage difference between strength generated in the two positions also significantly correlated with the quadriceps strength in the H45-K40 position: r_s_ = 0.41 (95%CI 0.19–0.58, *p* = 0.0002) and r_s_ = 0.54 (95%CI 0.35–0.68, *p*< 0.0001), respectively, in non-fixed and fixed dynamometer configuration.

Biases were also observed for the fixed dynamometer configuration when compared to non-fixed configuration (Figure 4), but these biases were less substantial: 1.23% (95% confidence interval agreement limit from −33.8–31.37%) and 0.3% (95% confidence interval agreement limit from −54.08–53.48%) in the fixed configuration, respectively, in the H45-K40 and the H90-K90 position. The percentage difference between quadriceps strength measured in fixed configuration and non-fixed configuration in the H45-K40 position was similar in the weakest subjects (strength < percentile 25) and in the strongest subjects (>percentile 75): respectively, 1.3 (−13.1–9.5)% and 3.4 (−2.5–11.9)% (*p* = 0.354). On the contrary, the percentage difference between quadriceps strength measured in fixed configuration and non-fixed configuration in the H90-K90 position was greater in strongest subjects compared to weakest subjects: respectively, −18.8 (−40.4–5.2)% and 2.5 (−15–12.2)% (*p* = 0.0462).

## 4. Discussion

This pragmatic study is a methodological focus on the effects of dynamometry conditions during quadriceps isometric contractions, as used in an ICU setting. It demonstrated that body position influenced the level of strength generated by the quadriceps. However, evaluation of isometric knee extension was not dependant on dynamometer configuration. These data are highly valuable for ICU clinicians and researchers to acquire reliable and consistent measures.

With all other factors of the dynamometry protocol identical, quadriceps strength generated in the seated (H90-K90) position was lower than the strength generated in the supine (H45-K40) position, without any impact of perceived discomfort in either position. This finding was not unexpected, as limb position is a known factor influencing strength measurement [22]. From a biomechanical point of view, variations in the related joints angle of a biarticular muscle (rectus femoris, part of the quadriceps muscle) changes the muscle length and in turn affects muscle force according to the length-tension relationship [23]. Effects of leg position on quadriceps strength measurement using an isometric contraction have previously been observed in a previous study comparing other supine and seated positions (respectively, H0-K35 and H90-K35) [24]. In addition to the length of the rectus femoris, other physiological and biomechanical factors probably determine the knee angle-torque relationship. These factors include the level of voluntary activation of the muscle, the agonist-antagonist co-contraction, and the changes in the length of all quadriceps femoris muscles according to their lever arm [25,26].

In the present study, the underestimation of strength in the H90-K90 position is not clinically negligible, reaching 27–28% of the strength measured in the H45-K40 position. Indeed, this bias is greater or equal to the minimal detectable changes of knee extension force measurement obtained with a handheld dynamometer in previously published studies performed in similar population or in older patients [19,27]. Nevertheless, the observed bias was greater in patients who generated greater quadriceps strength. In critically ill patients who are generally weaker than healthy subjects [20], such bias could thus be less important. However, it remains to be explored in this population.

The absence of clinically significant observed bias between non-fixed and fixed dynamometer configuration in the whole cohort is more surprising, as previous data seemed to indicate a higher measure reliability using a fixed dynamometer [24,28]. In the present study, the unique examiner was trained in the technique and the protocol, which could have minimised bias between the two configurations. It could be advisable to use a standardised fixed configuration in case of multiple examiners who differ in terms of proficiency and personal muscle strength. However, in the seated position, the difference between quadriceps strength measured in the two configurations was greater in the strongest subjects. This suggest that the fixed configuration should be favoured, at least in the seated position, in patients who can develop high levels of quadriceps strength (i.e., >percentile 75).

The present data could also be useful in clinical contexts other than critical illness, in which quadriceps strength is suitable for muscle weakness screening. This is typically the case in elderly patients, or in the post-operative period [29,30]. Indeed, surgery induces inflammation and changes in postoperative muscle performances. These modifications are still poorly investigated, particularly in older adults. Acquiring reliable data will be of importance for further research focusing on these topics [31].

## 5. Conclusions

Body position influenced the level of generated QS: the quadriceps was less efficient in the seated position, compared with the supine position, in healthy volunteers. On the contrary, the dynamometer configuration did not seem to impact the generated QS, except in the seated position in the strongest subjects. These findings have implications for further assessments in critically ill patients. The same position should be used for repeated QS measurements during a follow-up to allow intra-individual comparisons. When testing subjects who can develop high levels of quadriceps strength in the seated position, it should be preferable to use a fixed dynamometer configuration. Moreover, the testing position and configuration should be precisely documented in the method section of all related research reports.

## Figures and Tables

**Table 1:** Raw and relative quadriceps strength, and discomfort scores according to testing positions and dynamometer configurations. *p* value ^a^: *p* value related to comparisons between the two positions. *p* value ^b^: *p* value related to comparisons between the two dynamometer configurations.

	Quadriceps Strength (N)			Quadriceps Strength (N/kg)			Discomfort (NRS 0–10)		
	Supine (H45-K45)	Seated (H90-K90)	*p* Value ^a^	Supine (H45-K40)	Seated (H90-K90)	*p* Value ^a^	Supine (H45-K40)	Seated (H90-K90)	*p* Value ^a^
Non-fixed	428.4 [338.1–508.1]	307.4 [248.4–381.7]	<0.001	5.91 [4.86–7.38]	4.38 [3.40–5.65]	<0.001	2 [0–3]	1 [0–2.5]	0.009
Fixed	419.5 [289.6–525.4]	324.7 [244.2–375.5]	<0.001	5.83 [4.31–7.10]	4.46 [3.51–5.00]	<0.001	1 [0–3]	1 [0–3]	0.969
*p* value ^b^	0.727	0.875		0.912	0.664		0.471	0.052	

## Data Availability

Data supporting reported results can be presented upon reasonable request.

## References

[1] Rousseau A.F., Prescott H.C., Brett S.J., Weiss B., Azoulay E., Creteur J., Latronico N., Hough C.L., Weber-Carstens S., Vincent J.-L. (2021). Long-term outcomes after critical illness: Recent insights. Crit. Care.

[2] Hermans G., Van den Berghe G. (2015). Clinical review: Intensive care unit acquired weakness. Crit. Care.

[3] Parry S.M., Granger C.L., Berney S., Jones J., Beach L., El-Ansary D., Koopman R., Denehy L. (2015). Assessment of impairment and activity limitations in the critically ill: A systematic review of measurement instruments and their clinimetric properties. Intensive Care Med..

[4] Bittner E.A., Martyn J.A., George E., Frontera W.R., Eikermann M. (2009). Measurement of muscle strength in the intensive care unit. Crit. Care Med..

[5] Kress J.P., Hall J.B. (2014). ICU-acquired weakness and recovery from critical illness. N. Engl. J. Med..

[6] Bohannon R.W. (2005). Manual muscle testing: Does it meet the standards of an adequate screening test?. Clin. Rehabil..

[7] Vanpee G., Hermans G., Segers J., Gosselink R. (2014). Assessment of limb muscle strength in critically ill patients: A systematic review. Crit. Care Med..

[8] Ali N.A., O’Brien J.M., Hoffmann S.P., Phillips G., Garland A., Finley J.C., Almoosa K., Hejal R., Wolf K.M., Lemeshow S. (2008). Acquired weakness, handgrip strength, and mortality in critically ill patients. Am. J. Respir. Crit. Care Med..

[9] Piva S., Fagoni N., Latronico N. (2019). Intensive care unit-acquired weakness: Unanswered questions and targets for future research. F1000Research.

[10] Chan O.Y., van Houwelingen A.H., Gussekloo J., Blom J.W., den Elzen W.P. (2014). Comparison of quadriceps strength and handgrip strength in their association with health outcomes in older adults in primary care. Age.

[11] Bohannon R.W. (2009). Dynamometer measurements of grip and knee extension strength: Are they indicative of overall limb and trunk muscle strength?. Percept. Mot. Skills.

[12] Moxley Scarborough D., Krebs D.E., Harris B.A. (1999). Quadriceps muscle strength and dynamic stability in elderly persons. Gait Posture.

[13] Schilling B.K., Karlage R.E., LeDoux M.S., Pfeiffer R.F., Weiss L.W., Falvo M.J. (2009). Impaired leg extensor strength in individuals with Parkinson disease and relatedness to functional mobility. Parkinsonism Relat. Disord..

[14] Ploutz-Snyder L.L., Manini T., Ploutz-Snyder R.J., Wolf D.A. (2002). Functionally relevant thresholds of quadriceps femoris strength. J. Gerontol. A Biol. Sci. Med. Sci..

[15] Vanpee G., Segers J., Van Mechelen H., Wouters P., Van den Berghe G., Hermans G., Gosselink R. (2011). The interobserver agreement of handheld dynamometry for muscle strength assessment in critically ill patients. Crit. Care Med..

[16] Buckinx F., Croisier J.L., Reginster J.Y., Dardenne N., Beaudart C., Slomian J., Leonard S., Bruyere O. (2017). Reliability of muscle strength measures obtained with a hand-held dynamometer in an elderly population. Clin. Physiol. Funct. Imaging.

[17] Bohannon R.W. (1997). Reference values for extremity muscle strength obtained by hand-held dynamometry from adults aged 20 to 79 years. Arch. Phys. Med. Rehabil..

[18] Hogrel J.Y., Payan C.A., Ollivier G., Tanant V., Attarian S., Couillandre A., Dupeyron A., Lacomblez L., Doppler V., Meininger V. (2007). Development of a French isometric strength normative database for adults using quantitative muscle testing. Arch. Phys. Med. Rehabil..

[19] Rousseau A.F., Kellens I., Freycenon G., Dardenne N., Bruyere O., Damas P., Croisier J.L. (2018). Highly standardized quadriceps dynamometry of critically ill adults at bedside: A step towards individualized rehabilitation. Acta Anaesthesiol. Belg..

[20] Blanjean A., Kellens I., Misset B., Joris J., Croisier J.L., Rousseau A.F. (2020). Quadriceps strength in intensive care unit survivors: Variability and influence of preadmission physical activity. Aust. Crit. Care.

[21] van Melick N., Meddeler B.M., Hoogeboom T.J., Nijhuis-van der Sanden M.W.G., van Cingel R.E.H. (2017). How to determine leg dominance: The agreement between self-reported and observed performance in healthy adults. PLoS ONE.

[22] Keating J.L., Matyas T.A. (1996). The influence of subject and test design on dynamometric measurements of extremity muscles. Phys. Ther..

[23] Visser J.J., Hoogkamer J.E., Bobbert M.F., Huijing P.A. (1990). Length and moment arm of human leg muscles as a function of knee and hip-joint angles. Eur. J. Appl. Physiol. Occup. Physiol..

[24] Kim W.K., Kim D.K., Seo K.M., Kang S.H. (2014). Reliability and validity of isometric knee extensor strength test with hand-held dynamometer depending on its fixation: A pilot study. Ann. Rehabil. Med..

[25] Hahn D., Olvermann M., Richtberg J., Seiberl W., Schwirtz A. (2011). Knee and ankle joint torque-angle relationships of multi-joint leg extension. J. Biomech..

[26] Trezise J., Collier N., Blazevich A.J. (2016). Anatomical and neuromuscular variables strongly predict maximum knee extension torque in healthy men. Eur. J. Appl. Physiol..

[27] Bohannon R.W. (2012). Minimal detectable change of knee extension force measurements obtained by handheld dynamometry from older patients in 2 settings. J. Geriatr. Phys. Ther..

[28] Scott D.A., Bond E.Q., Sisto S.A., Nadler S.F. (2004). The intra- and interrater reliability of hip muscle strength assessments using a handheld versus a portable dynamometer anchoring station. Arch. Phys. Med. Rehabil..

[29] Fragala M.S., Alley D.E., Shardell M.D., Harris T.B., McLean R.R., Kiel D.P., Cawthon P.M., Dam T.T., Ferrucci L., Guralnik J.M. (2016). Comparison of Handgrip and Leg Extension Strength in Predicting Slow Gait Speed in Older Adults. J. Am. Geriatr. Soc..

[30] Lachmann G., Morgeli R., Kuenz S., Piper S.K., Spies C., Kurpanik M., Weber-Carstens S., Wollersheim T., Consortium B. (2020). Perioperatively Acquired Weakness. Anesth. Analg..

[31] Bautmans I., Van De Winkel N., Ackerman A., De Dobbeleer L., De Waele E., Beyer I., Mets T., Maggio M. (2014). Recovery of muscular performance after surgical stress in elderly patients. Curr. Pharm. Des..

